# Improving image quality and in-stent restenosis diagnosis with high-resolution “double-low” coronary CT angiography in patients after percutaneous coronary intervention

**DOI:** 10.3389/fcvm.2024.1330824

**Published:** 2024-07-23

**Authors:** Wenjie Wu, Hefeng Zhan, Yiran Wang, Xueyan Ma, Jiameng Hou, Lichen Ren, Jie Liu, Luotong Wang, Yonggao Zhang

**Affiliations:** ^1^ Department of Radiology, First Affiliated Hospital of Zhengzhou University, Zhengzhou, China; ^2^ CT Imaging Research Center, GE Healthcare China, Beijing, China

**Keywords:** coronary CT angiography, percutaneous coronary intervention, in-stent restenosis, deep learning image reconstruction algorithm, SnapShot Freeze 2

## Abstract

**Objective:**

This study aims to investigate the image quality of a high-resolution, low-dose coronary CT angiography (CCTA) with deep learning image reconstruction (DLIR) and second-generation motion correction algorithms, namely, SnapShot Freeze 2 (SSF2) algorithm, and its diagnostic accuracy for in-stent restenosis (ISR) in patients after percutaneous coronary intervention (PCI), in comparison with standard-dose CCTA with high-definition mode reconstructed by adaptive statistical iterative reconstruction Veo algorithm (ASIR-V) and the first-generation motion correction algorithm, namely, SnapShot Freeze 1 (SSF1).

**Methods:**

Patients after PCI and suspected of having ISR scheduled for high-resolution CCTA (randomly for 100 kVp low-dose CCTA or 120 kVp standard-dose) and invasive coronary angiography (ICA) were prospectively enrolled in this study. After the basic information pairing, a total of 105 patients were divided into the LD group (60 patients underwent 100 kVp low-dose CCTA reconstructed with DLIR and SSF2) and the SD group (45 patients underwent 120 kVp standard-dose CCTA reconstructed with ASIR-V and SSF1). Radiation and contrast medium doses, objective image quality including CT value, image noise (standard deviation), signal-to-noise ratio (SNR), and contrast-to-noise ratio (CNR) for the aorta, left main artery (LMA), left ascending artery (LAD), left circumflex artery (LCX), and right coronary artery (RCA) of the two groups were compared. A five-point scoring system was used for the overall image quality and stent appearance evaluation. Binary ISR was defined as an in-stent neointimal proliferation with diameter stenosis ≥50% to assess the diagnostic performance between the LD group and SD group with ICA as the standard reference.

**Results:**

The LD group achieved better objective and subjective image quality than that of the SD group even with 39.1% radiation dose reduction and 28.0% contrast media reduction. The LD group improved the diagnostic accuracy for coronary ISR to 94.2% from the 83.8% of the SD group on the stent level and decreased the ratio of false-positive cases by 19.2%.

**Conclusion:**

Compared with standard-dose CCTA with ASIR-V and SSF1, the high-resolution, low-dose CCTA with DLIR and SSF2 reconstruction algorithms further improves the image quality and diagnostic performance for coronary ISR at 39.1% radiation dose reduction and 28.0% contrast dose reduction.

## Key points

• Coronary computed tomography angiography with deep learning image reconstruction (DLIR) and SnapShot Freeze 2 (SSF2) technologies provides better diagnostic performance on in-stent restenosis (ISR).
• The scan protocol combining 100 kVp tube voltage and high-strength deep learning image reconstruction algorithm protocol can be used to significantly reduce radiation and contrast doses in coronary computed tomography angiography compared with the conventional protocol.
• The second-generation motion correction algorithm (SSF2) further reduces heart motion artifacts compared with the first-generation motion correction algorithm (SSF1) in CCTA.

## Introduction

Cardiovascular diseases (CADs) have gradually become one of the most critical factors threatening human health (1). Percutaneous coronary intervention (PCI), which could relieve coronary stenosis for myocardial revascularization with a high postoperative recanalization rate exceeding 90%, significantly improves the survival rates and quality of life for patients with CADs (2, 3). However, in-stent restenosis (ISR) remains one of the significant challenges for PCI. Although drug-eluting stents have significantly reduced the incidence of postoperative ISR compared with bare metal stents, nearly 10% of patients still suffer from ISR, which may be diagnosed by using the non-invasive coronary computed tomography angiography (CCTA) (4–6).

Various new imaging techniques are used to improve the spatial and temporal resolution in CCTA and to improve the visualization of the in-stent lumen and the diagnostic accuracy of ISR, such as wide-coverage CT detectors, high-resolution CT scan mode, motion correction algorithms, and image reconstruction algorithms (7, 8).

On the one hand, the metal artifacts introduced by the stents themselves could cause unclear stent boundaries that affect the observation of thrombus and calcified plaques on the vessel wall, which may cause the overdiagnosis of ISR. These metal artifacts may be reduced by using high tube voltages and high spatial resolution. On the other hand, the high spatial resolution required by CCTAs in patients with stents is still typically associated with the high radiation dosage, and the contrast medium dose is increased accordingly. Therefore, there is a need to reduce radiation dose and contrast dose while still maintaining high spatial resolution in imaging metal stents (9, 10).

Recently, new CT technologies, including the 230 μm high spatial resolution scan mode with focal-spot wabble (Hi-Res mode), the second-generation motion correction algorithm [SnapShot Freeze 2 (SSF2)], and the state-of-the-art deep learning image reconstruction (DLIR), have been introduced and integrated into a new CT scanner. Studies have shown that based on the deep convolutional neural network, DLIR could assist the “double-low” scan protocol (low radiation dose and contrast medium dose) while producing high-quality clinical CCTA images (11–13).

We thus hypothesize that the combination of these new CT technologies could be used to further improve image temporal and spatial resolution and reduce image noise at even reduced radiation and contrast medium doses. To our knowledge, few studies have been established to investigate the combination of the Hi-Res scan mode with SSF2 and DLIR algorithm on radiation and contrast dose reduction for CCTA in patients after PCI. Therefore, in this study, we assessed the image quality and accuracy in diagnosing ISR using “double-low” CCTA reconstructed with DLIR and SSF2 compared with standard-dose CCTA under high-definition reconstruction mode with volume-based adaptive statistical iterative reconstruction (ASIR-V) algorithm and first-generation motion correction algorithm (SSF1).

## Materials and methods

### Study population

**Figure 1:**
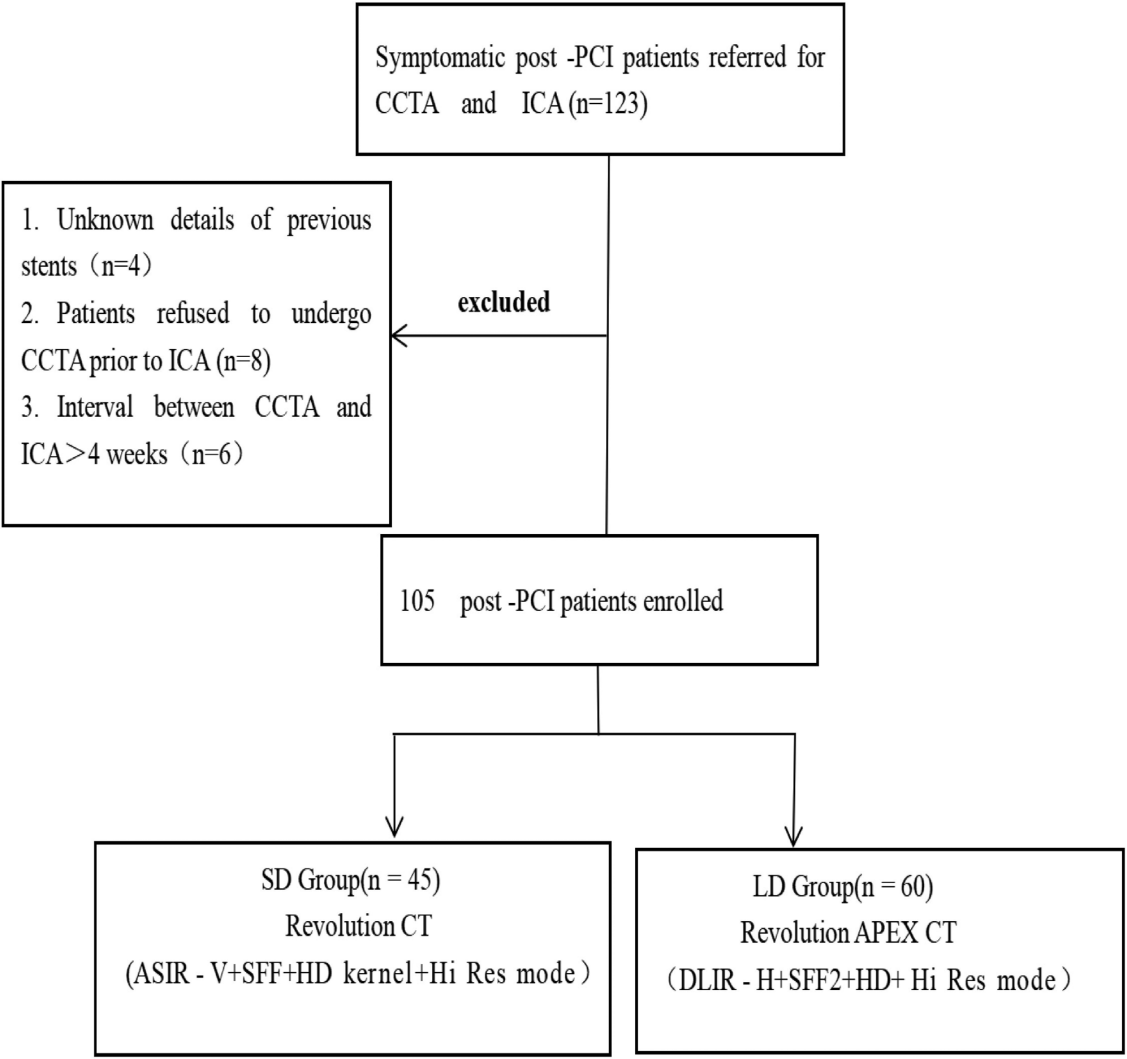
Flowchart of the study design. CCTA, coronary computed tomography angiography; ICA, invasive coronary angiography; PCI, percutaneous coronary intervention; DLIR-H, high-strength deep learning image reconstruction algorithm; ASIR-V, adaptive statistical iterative reconstruction Veo algorithm; Hi-Res mode, high-resolution mode; HD kernel, high-definition kernel.

This prospective study was approved by the ethics committee of our hospital, and all patients provided informed consent. A total of 123 patients who had undergone PCI with drug-eluting stents and who were scheduled for CCTA (randomly underwent low-dose CCTA or standard-dose CCTA) and invasive coronary angiography (ICA) from January 2022 to July 2022 were prospectively enrolled (Figure 1). The inclusion criteria included patients with prior PCI and implantation of a drug-eluting stent and those undergoing CCTA and ICA for suspected in-stent restenosis. The exclusion criteria included the following: (a) iodine contrast agent allergy; severe liver and kidney insufficiency; (b) decompensated cardiac insufficiency; (c) absence of ICA examinations or a CCTA and ICA interval longer than 1 month; and (e) patients with stent-in-stent or missing stent records (14). Finally, 105 patients were included in the study and were divided into the low-dose group (LD group) and standard-dose group (SD group) according to the CCTA protocols.

### CCTA acquisition

An oral and/or intravenous beta-blocker or non-dihydropyridine calcium channel was administered to patients presenting with a heart rate (HR) above 70 beats per minute to lower it below 70 beats per minute. However, in this subset of selected patients, further therapy was limited if it was deemed clinically unsafe to do so, based on symptoms such as lightheadedness or dyspnea or concerns of hypotension.

All scans were acquired in high-resolution mode (Hi-Res mode) with an in-plane spatial resolution of 230 μm, a layer thickness and an interval of 0.625 mm, a tube rotation time of 0.28 s, and a reconstruction matrix of 512 × 512. The patients in the low-dose group (LD group) were examined on a new 16 cm wide-detector CT scanner (Revolution Apex, GE HealthCare). The other scanning parameters for the LD group with Apex CT were as follows: tube voltage, 100 kVp; noise index, 36 HU; and tube current automatically adjusted between 500 mA and 1,300 mA. Images were reconstructed with high-level DLIR under the high-definition kernel and the second-generation SnapShot Freeze algorithm for cardiac motion correction [SnapShot Freeze 2 (SSF2), GE HealthCare]. The patients in the standard-dose group (SD group) were examined on a 16 cm wide-detector CT scanner (Revolution CT, GE HealthCare). The other scanning parameters for the SD group with Revolution CT were as follows: tube voltage, 120 kVp; noise index, 26 HU; and tube current automatically adjusted between 100 mA and 720 mA. Images were reconstructed with ASIR-V with 50% weight, and the first-generation SnapShot Freeze (SSF1, GE HealthCare) algorithm for cardiac motion correction (Table 1).

**Table 1:** Scanning parameters.

	SD group (*n* = 45)	LD group (*n* = 60)
Scan mode	Hi-Res mode	Hi-Res mode
Detector configuration (mm)	256 × 0.625 mm	256 × 0.625 mm
Rotation time (s)	0.28	0.28
Tube potential (kVp)	120	100
Tube current modulation range (mA)	100–720	500–1,300
Noise index (NI, HU)	26	36
Reconstruction kernel	HD kernel	HD kernel
Reconstruction algorithms	ASIR-V 50%	DLIR-H
Motion correction algorithms	SSF1	SSF2

SD group, standard-dose group; LD group, low-dose group; SSF1, SnapShot Freeze 1; SSF2, SnapShot Freeze 2; HD kernel, high-definition kernel; Hi-Res mode, high-resolution mode.

All CCTA scans used the prospective ECG-triggered axial scan mode. The *z*-axis coverages were chosen to be 120 mm, 140 mm, or 160 mm based on the heart size of different patients to optimize dose performance. The scan range for both groups covered 1 cm below the tracheal ridge to about 1 cm below the apex of the heart. According to the patient's heart rate before scanning, the auto-gating technique was used to select the ideal cardiac phases. For patients whose heart rates were <66 bpm, the exposure time window was 70%–80% of the RR interval; for patients with HR >86 bpm, the exposure time window was 40%–55% of the RR interval. Images of the whole heart would be acquired in one heartbeat for patients with an irregular heart rate (heart rate variability over 10 beats/min).

The patients were placed in the supine position for both groups, and sublingual nitroglycerin (0.5 mg) was administered to the patients before scanning to dilate the coronary arteries. Using the intelligent automatic tracking technology for the contrast agent, the trigger point was placed in the descending aorta (1 cm below the tracheal bifurcation), the trigger threshold was set to 150 HU, and the image acquisition started with a delay of 2 s after the threshold was reached. All patients in both LD and SD groups were injected through the right anterior elbow veins using a double-tube syringe with a non-ionizing contrast agent (ultrafast injection, concentration of 370 mgI/ml, Bayer Medicine BAYX). Regarding the contrast injection protocol, the LD group used a low-contrast medium protocol with a weight-dependent contrast dose rate of 18 mgI/kg/s, while the SD group used 32 mgI/kg/s. Both groups used 10 s injection time, and the flow rate for contrast injection was determined by dividing the total contrast medium volume by the 10 s injection time. For example, in the LD group, for patients with a body weight of 50 kg, the total specific volume would be (18 mgI/kg/s × 50 kg × 10 s)/(370 mg/ml) = 24 ml, and the flow rate would be 24 ml/10 s = 2.4 ml/s. After the injection of the contrast medium, both groups were injected with 30 ml of normal saline at the same flow rate.

### Post-processing and image quality evaluation

For the objective evaluation, the axial images were transferred to the AW 4.7 Workstation, and all patient information was made anonymous. The maximum intensity projection (MPR), volume rendering (VR), maximum intensity projection (MIP), and curved planar reformat (CPR) images were then generated for image post-processing. Two radiologists both with 5 years of experience in CCTA conducted the objective assessment. A region of interest (ROI) of 2 mm diameter was used for coronary artery branches, avoiding vessel walls, plaques, and stenosis as much as possible. The ROIs for both groups were rigorously kept in the same size and placed in a similar place for both groups.

The CT attenuation value and image noise value in HU of the aorta, left main artery (LMA), left ascending artery (LAD), left circumflex artery (LCX), and right coronary artery (RCA) in the two groups were recorded and compared. The signal-to-noise ratio (SNR) and contrast-to-noise ratio (CNR) were calculated as follows:

$$\mathrm{SN}R_{\mathrm{vessel}}=\frac{\mathrm{CT}\;\mathrm{valu}e_{\mathrm{vessel}}}{\mathrm{Image}\;\mathrm{Nois}e_{\mathrm{vessel}}}$$

$$\mathrm{CN}R_{\mathrm{vessel}}=\frac{\mathrm{CT}\;\mathrm{valu}e_{\mathrm{vessel}}-\mathrm{CT}\;\mathrm{valu}e_{\mathrm{adjacent}\;\mathrm{epicardial}\;\mathrm{fat}}}{\mathrm{Image}\;\mathrm{Nois}e_{\mathrm{vessel}}}$$

where CT value_vessel_ is the mean CT value in HU of an ROI placed in the proximal coronary artery, Image Noise_vessel_ is the mean image noise value in HU of an ROI placed in the proximal coronary artery, and CT value_adjacent epicardial fat_ is the mean CT value in HU of the adjacent epicardial fat tissue.

### Subjective evaluation of the stents

The readers were blinded to clinical and group information. Two other radiologists, both with more than 8 years of experience in cardiovascular imaging, independently conducted the subjective evaluation of the images from the two groups using a five-point Likert scale for both the overall image quality and appearance of stents. For the overall image quality assessment: 5 = excellent (very little image noise and extremely readable images); 4 = good (good image quality without diagnostic limitation); 3 = acceptable (usual images in image interpretation); 2 = suboptimal (with some diagnostic limitation), and 1 = unacceptable (massive image noise and scarcely readable images). For the appearance of stent assessment: 5 = excellent (the edges of the stents are sharp, excellent attenuation of the vessel lumen, and clear delineation of the vessel walls); 4 = good (clear stent strut definition, minimal blooming artifacts from the stent); 3 = acceptable (minimal stent margin definition, some blooming artifacts); 2 = suboptimal (major artifacts affecting visualization of major structures of the stents), and 1 = unacceptable (poor vessel wall definition and severe artifacts). Images with a score of >2 were considered diagnostically acceptable (15).

### ICA inspection

We used the ISR diagnosis by invasive coronary angiography (ICA) within 30 days as the standard reference to assess the diagnostic performance of CCTA on ISR. Binary ISR is defined as stenosis ≥50% in the stent or within 5 mm of both ends of the stent (14). We have identified stenosis ≥50% in the stents or within 5 mm of both ends of the stents on CCTA and then assessed accuracy, sensitivity, specificity, positive predictive value (PPV), and negative predictive value (NPV) for binary ISR of the LD and SD groups on patient level and stent level. Accuracies were further analyzed for large-caliber stents (diameter, ≥3 mm) and small-caliber stents (diameter, <3 mm).

### Radiation dose and contrast dose

CT dose index (CTDI_vol_) and dose length product (DLP) were recorded after the scanning, and the effective dose was calculated as ED = *k* × DLP, where *k* = 0.014 mSv/mGy cm. Meanwhile, the contrast medium dose was also calculated and recorded.

### Statistical analysis

The SPSS (v 21.0, IBM Corp.) statistical analysis software was used for statistical analysis. The normality of measurement data was tested using the Kolmogorov–Smirnov test. Measurement data that conformed to normal distribution were expressed as mean ± standard deviation, and the count data were expressed as frequencies and percentages. The normal distribution variables between the two groups were compared using the two independent samples Student's *t*-test. The Mann–Whitney test was used to compare groups of measurement data that did not obey the normal distribution. The chi-square test or Fisher’s exact test was used for the categorical variables for the two groups. A *p*-value of <0.05 was considered statistically significant.

## Results

### Baseline characteristics

A total of 123 patients underwent CCTAs, and 105 patients with 231 stents were eligible for enrollment, including 32 males and 73 females, aged 38–79 years, with an average age of 64.7 ± 10.9 years. There were no clinical differences between the LD and SD groups. Sixty patients in the LD group had a total of 138 stents implanted, and there were 53 stents with a diameter of ≥3 mm and 40 stents with a diameter of <3 mm. Forty-five patients in the SD group had a total of 93 stents implanted, and there were 81 stents with a diameter of ≥3 mm and 57 stents with a diameter of <3 mm. There was no significant difference in stent position between the two groups (Table 2).

**Table 2:** Demographic and basic data.

	SD group (*n* = 45)	LD group (*n* = 60)	*T*/*χ*^2^ value	*p*-value
Age (years)	65.5 ± 9.124	63.83 ± 11.80	−0.832	0.424
Female/male	12/33	20/40	0.539	0.463
Average BMI, kg/m^2^	24.3 ± 5.29	23.6 ± 6.86	0.574	0.452
Heart rate, bpm	72.88 ± 29.42 70.0 [60.0, 86.0]	70.22 ± 34.0 67.0 [61.0, 75.0]	0.980	0.319
Risk factors				
Hyperlipidemia	5 (11.1%)	13 (21.6%)	2.712	0.100
Hypertension	32 (71.1%)	40 (66.6%)	0.236	0.627
Smoking	34 (75.5%)	25 (41.6%)	11.997	< 0.001
Diabetes	30 (66.6%)	18 (30%)	13.931	< 0.001
Clinical presentations			0.019	0.890
Stable angina	4 (8.8%)	7 (11.6%)	–	–
Unstable angina	41 (91.2%)	53 (88.4%)	–	–
Number of stents	93 (2.07/patients)	138 (2.3/patients)		
LMA	1 (1.07%)	3 (2.17%)	0.013	0.910
LAD	33 (35.4%)	55 (39.8%)	0.450	0.502
LCX	29 (31.18%)	34 (24.6%)	1.200	0.273
RCA	28 (30.1%)	45 (32.6%)	0.161	0.688
D1	2 (2.15%)	1 (0.72%)	0881	0.348
Stent diameters			0.066	0.892
≥3.0 mm	53 (56.9%)	81 (58.6%)	–	–
<3.0 mm	40 (43.1%)	57 (41.4%)	–	–
CTDIvol (mGy)	7.87 ± 0.54	4.92 ± 0.74	−19.932	< 0.001
DLP (mGy•cm)	117.43 ± 3.53	71.55 ± 4.53	−49.795	< 0.001
effective dose, ED (mSv)	1.64 ± 0.49	1.00 ± 0.64	−49.79	< 0.001
Tube current(mA)	574.34 ± 45.90	893.85 ± 119.47	15.5	< 0.001
Volume of contrast media (ml)	48.25 ± 7.45	34.74 ± 5.77	−9.677	< 0.001

*S*D group, standard-dose group; LD group, low-dose group; BMI, body mass index; LMA, left main artery; RCA, right coronary artery; LAD, left anterior descending branch; LCX, left circumflex branch; D1 diagonal branches; CTDIvol, volumetric CT dose index; DLP, dose length product; ED, effective dose; mSv, millisieverts. Category data are expressed as numbers with percentages in parentheses, with *χ*^2^. Parametric continuous data are expressed as means ± standard deviation (SD)s with *T* value. Heart rate data are expressed as means ± standard deviation (SD)s and median (25% quantile, 75% quantile).

### Radiation dose and contrast dose

Compared with the SD group, the LD group had a 39.0% reduction in effective dose (1.00 ± 0.64 mSv vs. 1.64 ± 0.49 mSv, *p* < 0.001). Notably, compared with the SD group, the tube current of the LD group was increased by 35.7% (893.85 ± 119.47 mA vs. 574.34 ± 45.90 mA, *p *< 0.001) due to the use of lower tube voltage. Meanwhile, the contrast dose was also significantly reduced in the LD group with a reduction of 28.0% (34.74 ± 5.77 ml vs. 48.25 ± 7.45 ml, *p* < 0.001) (Table 2).

### Image quality evaluation

**Figure 2:**
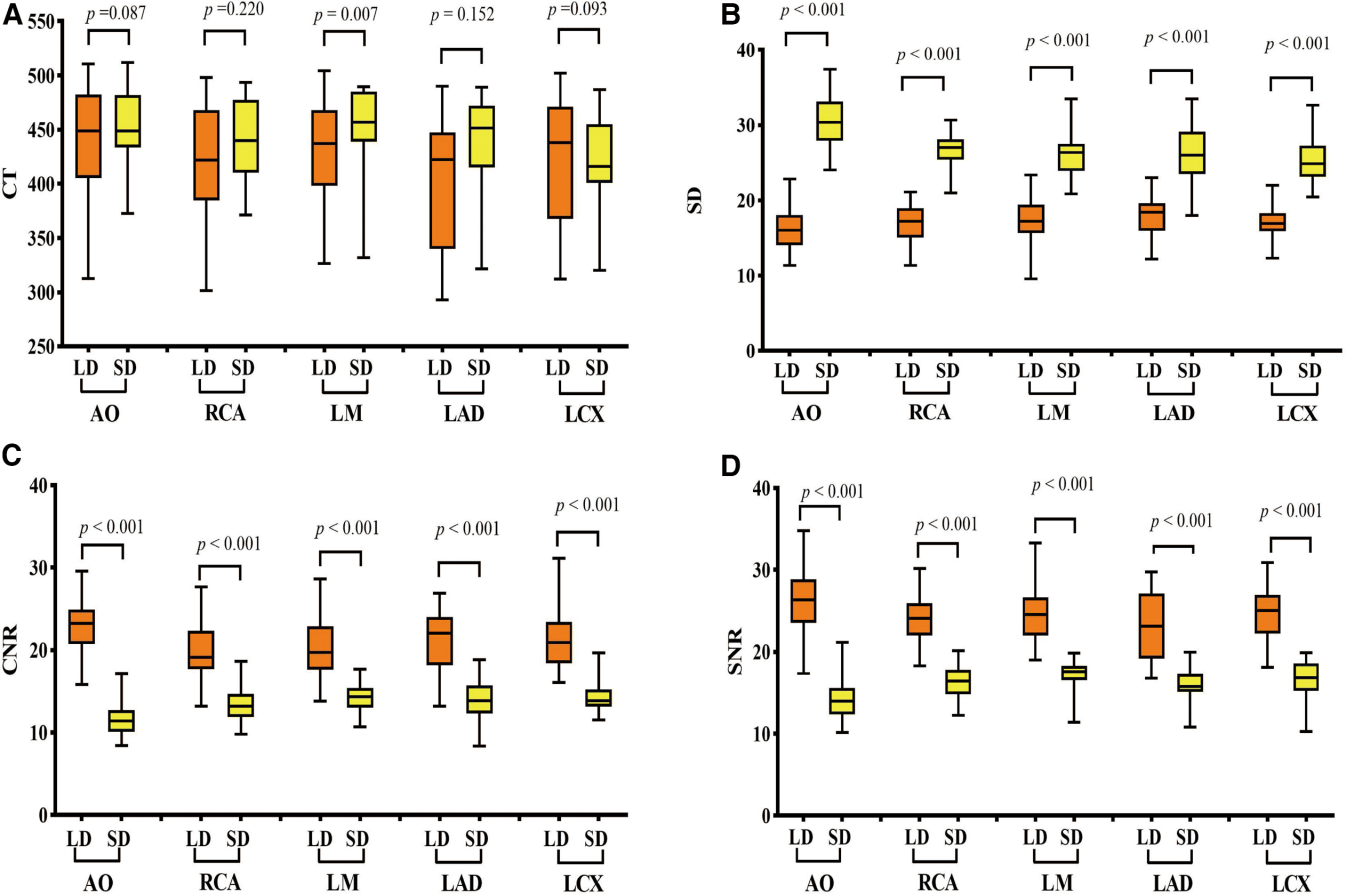
Comparison of objective image quality scores of vessels between the two groups. (**A**) CT values, (**B**) image noise values, (**C**) SNR values, and (**D**) CNR value comparison between the LD and HD groups. LD stands for low-dose group and HD stands for high-definition group. SNR, signal-to-noise ratio; CNR, contrast-to-noise ratio; AO, aortic root; RCA, right coronary artery; LMA, left main artery; LAD, left anterior descending branch; LCX, left circumflex branch. *p *< 0.001 was a significant difference.

**Figure 3:**
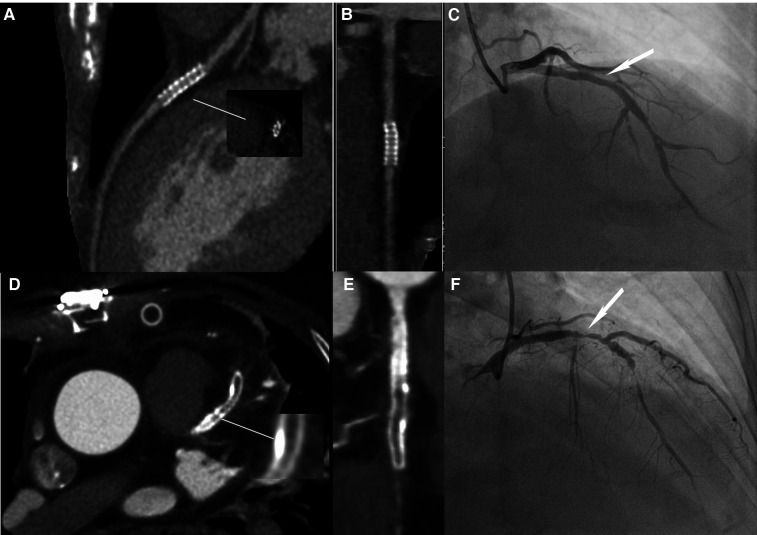
Image quality comparison between the LD and HD groups. (**A**–**C**) A 63-year-old male patient in the HD group, with a BMI of 24.53 kg/m^2^. (**A**) The image quality of CCTA reached a subjective score of 3: with minimal stent margin definition, some blooming artifacts, acceptable diagnostic information, and an image noise of 37.7 HU. (**B**) CPR image of LCX showed the absence of ISR in a 2.5 mm coronary stent (resolute, 2.5 × 21 mm, JHLD). (**C**) ICA of LCX confirmed the absence of ISR (white arrow). (**D**–**F**) A 61-year-old male patient in the LD group with a BMI of 24.06 kg/m^2^. (**D**) The image quality of CCTA reached a subjective score of 5: clear stent strut definition with a clear border of the blood vessel and an image noise of 16.7 HU. (**E**) CPR image of LAD showed focal ISR in a 3.0 mm coronary stent (resolute, 4.0 × 33 mm, RDES). (**F**) ICA of left anterior descending the diagnosis of focal ISR (white arrow). CCTA, coronary computed tomography angiography; CPR, curved planar reformation; ICA, invasive coronary angiography; ISR, in-stent restenosis; LCX, left circumflex; LAD, left anterior descending.

There was no significant difference in CT values at AO, RCA, LAD, and LCX between the LD and SD groups. The image noise values in all vessels of the LD group were significantly lower than those of the SD group, and the SNR and CNR values in all vessels of the LD group were significantly higher than those of the SD group (all *p *< 0.001) (Figure 2). The subjective scores of both overall image quality and stent appearance of the LD group were better than those of the SD group. CT image quality was at least good in almost all patients, and 99.05% (104/105) of patients were scored over 3 (Table 3 and Figure 3).

**Table 3:** Subjective image quality comparison.

Quality score	SD group (*n* = 45)	LD group (*n* = 60)
Overall image quality		
1	0	0
2	1	0
3	13	7
4	21	42
5	0	11
Stent appearance		
1	0	0
2	0	1
3	21	15
4	11	30
5	3	14

SD group, standard-dose group; LD group, low-dose group.

### Diagnostic performance for ISR

There were 93 stents in 45 patients (2.07 per patient) for the SD group and 138 stents in 60 patients (2.30 per patient) for the LD group. The average stenosis values are listed in Table 4.

**Table 4:** Comparison of the stenosis levels.

	SD group	LD group
Total stents (*n* = 231)	93	138
ICA (%)	23.66 ± 28.96	23.58 ± 28.30
CCTA (%)	25.43 ± 28.80	26.46 ± 29.74
ISR (*n* = 61)^a^	25	36
ICA (%)	68.91 ± 14.38	65.14 ± 18.65
CCTA (%)	67.39 ± 14.84	69.31 ± 16.26
Non-ISR (*n* = 170)^a^	68	102
ICA (%)	8.79 ± 12.05	8.76 ± 11.12
CCTA (%)	11.64 ± 15.99	11.19 ± 14.61

SD group, standard-dose group; LD group, low-dose group; ISR, in-stent restenosis.

^a^ Stents diagnosed as ISR or ruled out ISR by ICA.

Compared with the SD group, the LD group had better diagnostic performance in both patient-level and stent-level analyses. Specifically, the accuracy of ISR diagnosis was 94.2% for the LD group and 83.8% for the SD group, and PPV of ISR diagnosis was 81.8% for the LD group and 62.5% for the SD group (all *p *< 0.05) (Table 5). There were 23 cases, 15 cases in the SD group (3 in RCA, 8 in LAD, and 4 in LCX) and 8 cases (1 in RCA, 5 in LAD, and 3 in LCX) in the LD group, in which stenoses were detected only at CCTA but not at ICA.

**Table 5:** Comparison of the diagnostic performance on ISR.

	SD group	LD group	*p*-value
Patient-based analysis^a^			
*N*	45	60	
Accuracy	93.3% (42/45)	95.0% (57/60)	0.716
Sensitivity	100% (39/39)	100% (51/51)	1
Specificity	57.1% (4/7)	66.7% (6/9)	0.696
PPV	92.7 (39/42)	94.4 (51/54)	0.750
NPV	100%	100%	1
Stent-based analysis			
*N*	93	138	
Accuracy	83.8% (78/93)	94.2% (130/138)	0.010
Sensitivity	100% (25/25)	100% (36/36)	1
Specificity	77.9% (53/68)	92.1% (94/102)	0.080
PPV	62.5% (25/40)	81.8% (36/44)	0.047
NPV	100% (68/68)	100% (102/102)	1
Large-caliber stents (stent diameter, ≥3 mm)			
*N*	53	81	
Accuracy	92.4% (49/53)	98.7% (80/81)	0.059
Sensitivity	100% (18/18)	100% (27/27)	1
Specificity	88.5% (31/35)	98.1% (53/54)	0.055
PPV	81.8% (18/22)	96.4% (27/28)	0.087
NPV	100% (31/31)	100% (53/53)	1
Small-caliber stents (stent diameter, <3 mm)			
*N*	40	57	
Accuracy	72.5% (29/40)	87.7% (50/57)	0.058
Sensitivity	100% (6/6)	100% (9/9)	1
Specificity	67.6% (23/34)	85.4% (41/48)	0.055
PPV	35.2% (6/17)	56.2% (9/16)	0.022
NPV	100% (23/23)	100% (41/41)	1

SD group, standard-dose group; LD group, low-dose group; ISR, in-stent restenosis; NPV, negative predictive value; PPV, positive predictive value.

^a^ ISR and native obstructive coronary lesions were both included for patient-based analysis.

For ISR diagnostic performance on the large-caliber stents, the PPV of the LD group was nearly 10% higher than that of the SD group. For ISR diagnostic performance on the small-caliber stents, the PPV of the LD group was nearly 20% higher than that of the SD group (Table 5).

## Discussion

In this study, by comparing the low-dose CCTA with DLIR and SSF2 with standard-dose CCTA with ASIR-V and SSF1, we demonstrated that the low-dose CCTA with DLIR and SSF2 could further improve the image quality and diagnostic performance for coronary ISR in reduced radiation dose and contrast medium dose conditions. As far as we know, this was the first clinical study to investigate the radiation dose and contrast dose saving abilities of DLIR in a Hi-Res model in combination with a motion correction algorithm.

Multiple CCTA studies have confirmed that images reconstructed with DLIR have better image quality than hybrid iterative reconstructions (16–18). Furthermore, some “double-low” studies have shown the ability of radiation dose and contrast dose saving for CCTA for patients without stents. In our study, we further extended the study scope by establishing a “double-low” CCTA protocol in combination with DLIR and SSF2 algorithms for patients after PCI. Our study proved that CCTA reconstructed with DLIR and SSF2 could reduce 39.1% radiation dose and 28% contrast dose while maintaining the image quality and diagnostic performance compared with CCTA with ASIR-V 50% and SSF (19, 20).

Moreover, we proved that the “double-low” CCTA with DLIR and SSF2 outperformed the standard-dose CCTA in the diagnosis of ISR with ICA as the reference (21). It is known that CCTA under high-resolution mode is suitable for patients with many calcified lesions or after PCI, which can effectively reduce the interference of metal artifacts and make the lumen of coronary stents more clearly displayed. However, the increase in image noise could make it more challenging to display the small intimal hyperplasia and stenosis in stents, especially in stents with a diameter of ≤3 mm (22), making the diagnosis potentially less accurate. Studies have utilized modified subtraction CCTA imaging techniques (23), iterative algorithms (24), or imaging markers [such as fractional flow reserve (25) and perivascular fat attenuation index (26)] to improve visualization of coronary ISR. In our study, we assessed the combination of the most advanced CT technologies for CCTA in patients with stents, including 16 cm wide-coverage detectors with 230 μm spatial resolution and high temporal resolution assisted by the new-generation motion correction algorithm SSF2 and DLIR. Eckert et al. (27) found that nearly two-thirds of symptomatic patients after PCI could rule out ISR by CCTA, with 99% NPV and 75% PPV. In our study, the NPV and PPV for the LD group were 100% and 94.4%, respectively. Li et al. (14) used the third-generation dual-source CT (SOMATOM Force) combined with low-dose imaging to accurately diagnose coronary ISR with large-bore (≥3 mm) and small-bore (<3 mm) stents. The diagnostic accuracy of ISR of small-bore and large-bore stents were 88.5% and 98.7% with 1.30 ± 0.72 mSv effective dose and 50 ml contrast media. In our study, the diagnostic accuracy of ISR of small-bore and large-bore stents were 87.7% and 98.7% with a reduced effective dose of 1.00 ± 0.64 mSv and contrast medium dose of 34.74 ± 5.77 ml in the LD group. Notably, the patient heart rate range during acquisition in the study of Li et al. was 63–83 bpm, while the heart rate of the patients in the LD group during acquisition in our study was 50–93 bpm. We suggest both the study of Li et al. (14) and our study have testified the clinical benefits of the new CT technologies in CCTA for patients with ISR and provided essential high-quality clinical information for follow-up after PCI, which is of great significance for improving curative effect and prognosis.

**Figure 4:**
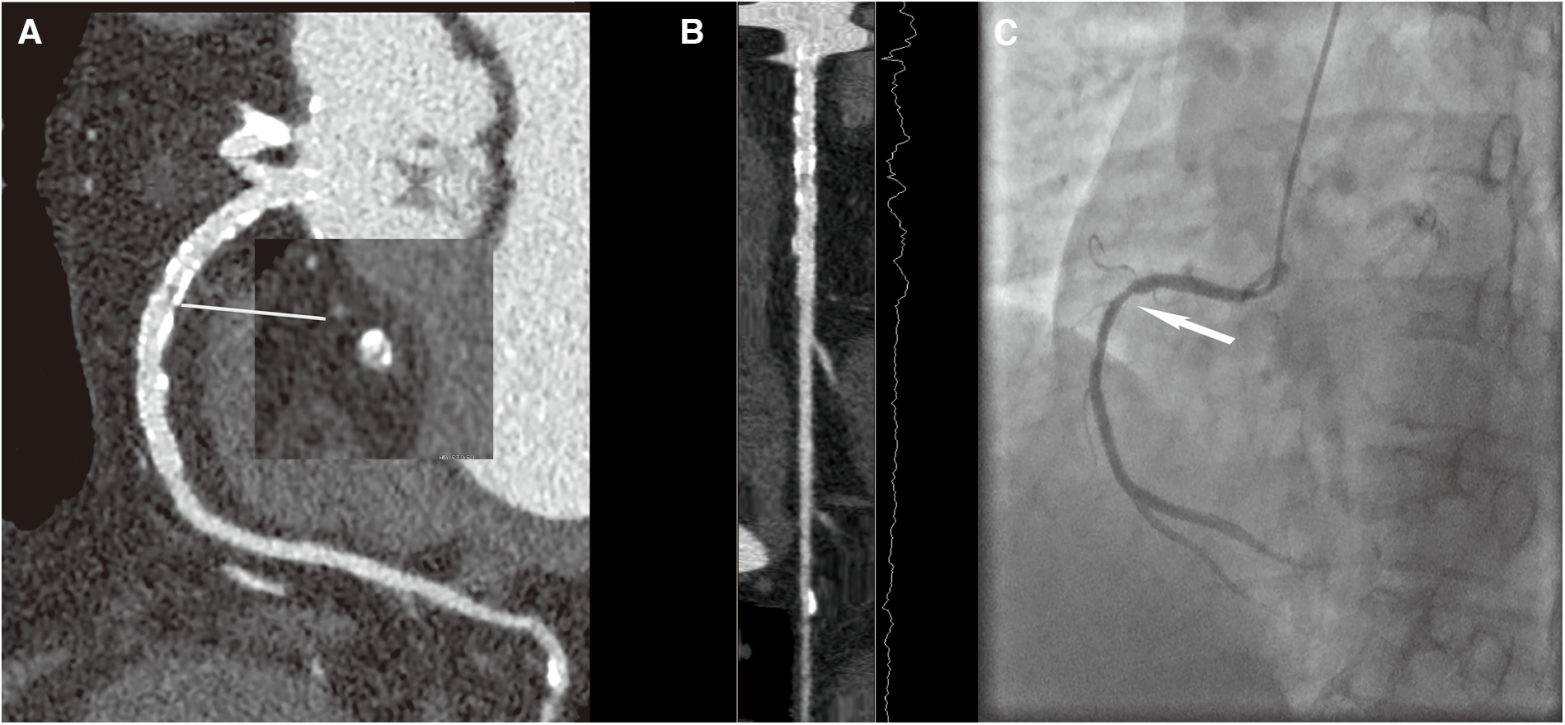
A 70-year-old female patient with ISR in the HD group, with a BMI of 26.4 kg/m^2^. (**A**) The image quality reached a subjective score of 4 with minimal stent margin definition, some blooming artifacts, acceptable diagnostic information, and an image noise of 35 HU. (**B**) CPR image of RCA showed focal ISR in a 3.0 mm coronary stent (resolute 3.0 × 33 mm, Firebird). (**C**) ICA of the right coronary artery confirmed the diagnosis of focal ISR (white arrow).

**Figure 5:**
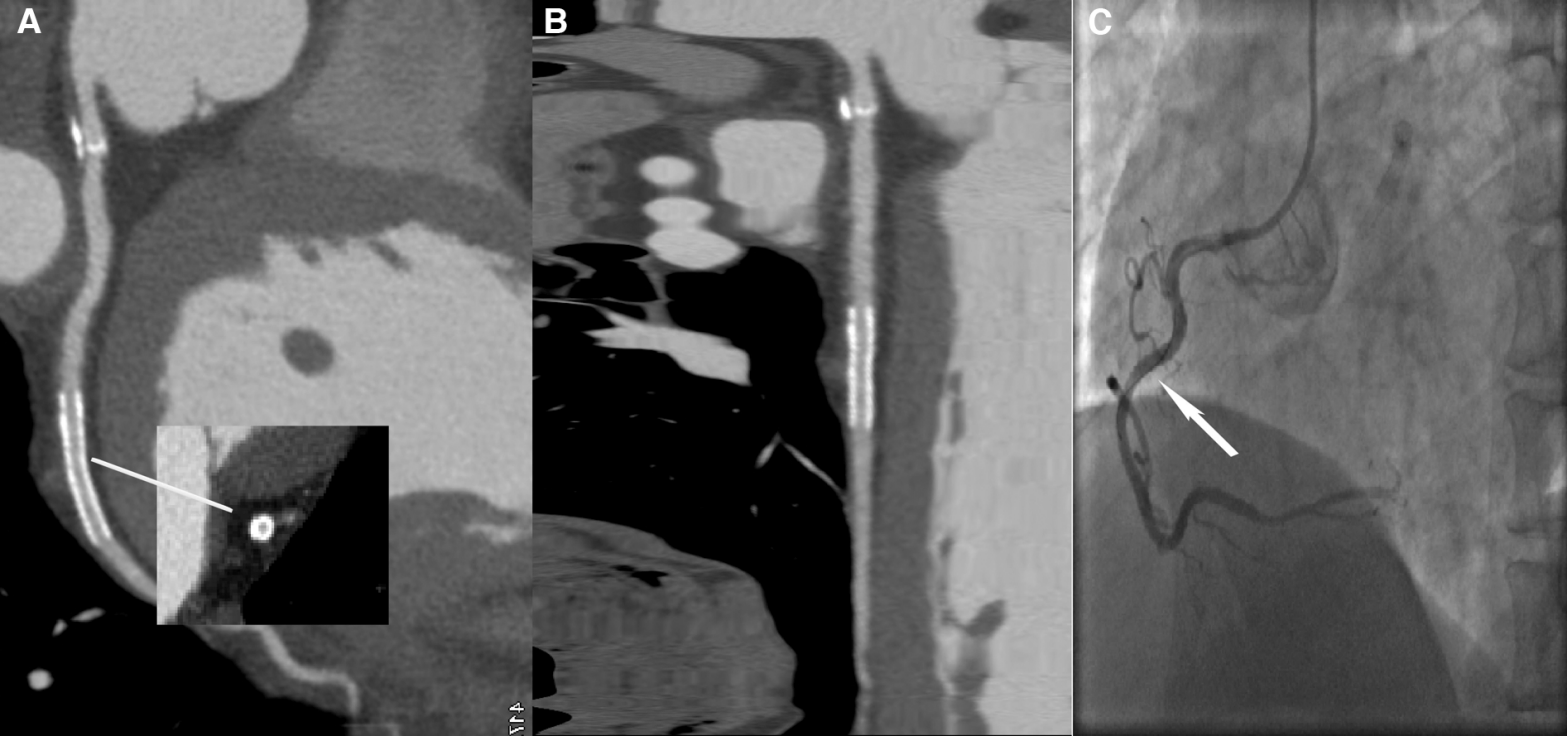
A 59-year-old male patient with ISR in the LD group, with a BMI of 25.56 kg/m^2^. (**A**) The image quality reached a subjective score of 5: clear stent strut definition with a clear border of the blood vessel and an image noise of 16 HU. (**B**) CPR image of LCX showed the absence of ISR in a 2.5 mm coronary stent (resolute, 2.5 × 22 mm, XIENCE Alpine). (**C**) ICA of the left circumflex confirmed the stent patency (white arrow).

Previous studies have shown that CCTA was the first-line non-invasive imaging technique in patients with suspected coronary artery disease and ICA remained the standard reference method for the identification and characterization of coronary artery stenosis. When anatomical coronary narrowing >50% on ICA was considered a reference standard, CCTA had a specificity of 78%. CCTA with high-resolution CT could achieve a specificity of 88% in a per-vessel analysis compared with ICA in patients with high calcium scores provided promising preliminary data demonstrating a high diagnostic accuracy with a specificity of 88% in a per-vessel analysis compared with ICA (28, 29). In our study, in agreement with previous studies, the specificity values were 77.9% for the SD group and 94.2% for the LD group in a per-vessel analysis compared with ICA for patients after PCI. Our results were coordinated with the finding of the previous study that the high-resolution mode CCTA protocol could decrease the false-positive findings than the traditional mode CCTA (28). Furthermore, we have noticed that the diagnostic accuracy and PPV for ISR in the LD group were higher than those in the SD group, which proved that compared with the traditional iterative reconstruction in combination with SSF1, CCTA images of DLIR and SSF2 technologies had better diagnostic performance on ISR with better noise containment and motion correction capability. Specifically, the accuracy and PPV of ISR diagnosis were 10.4% and 19.3% higher for the LD group than those for the SD group. We further reviewed the misdiagnosed 23 cases, 15 (4 for the large-caliber stent subgroup and 11 for the small-caliber stent subgroup) were misdiagnosed in the SD group, and only 8 (1 for the large-caliber stent subgroup and 7 for the small-caliber stent subgroup) were misdiagnosed in the LD group. We noticed that the misdiagnosed rates of the SD group were higher than those of the LD group. We have found that all the misdiagnosed 23 cases were overestimation of luminal stenosis, which we speculate is the combination of heart motion, excessive calcium plaque load, small stent size, and particularly the still limited spatial resolution of the CT systems. However, the fact that the LD group had a much lower misdiagnosis rate and that the LD group with SSF2 provided much better performance on the visibility of luminal stenosis (Figures 4, 5) demonstrated the importance of motion correction in providing accurate IRS diagnosis for patients after PCI.

This study has several limitations. First, all images were acquired on two CT scanners from the same vendor to minimize systemic errors. Therefore, the applicability of these findings to scans obtained in other types of scanners from different vendors may be limited. Second, we only compared ASIR-V 50% and DLIR-H in our study. We noticed that 50% was the most used iterative reconstruction algorithm and DLIR-H provided the highest potential on radiation dosage and contrast medium dosage reduction among the three levels of DLIR. In the future, we plan to perform further studies on the comprehensive comparison of all levels of ASIR-V and DLIR algorithms on patients after PCI. Finally, we noticed that all the misdiagnosed 23 cases had overestimation on the degree of luminal stenosis. Further study should establish subgroup analysis to find out the dominant factors for the overestimation.

## Conclusion

In summary, as the more advanced reconstruction methods, DLIR and SSF2 could further significantly reduce image noise and cardiac motion artifacts, respectively, compared with ASIR-V and SSF1 reconstruction algorithms. The combination of DLIR and SSF2 in CCTA for patients with PCI could effectively reduce the radiation and contrast medium dose while improving the quality of CCTA images and providing high diagnostic efficiency for ISR.

## Data Availability

The data that support the findings of this study are available, requests to access the datasets should be directed to WW at 244171013@qq.com.
